# Traumatic bony avulsion of the lateral collateral ligament of the knee in a pediatric patient

**DOI:** 10.1093/jscr/rjag790

**Published:** 2026-09-14

**Authors:** Julian Ramin Andresen, Stephan Payr, Stefan Hajdu, Silke Aldrian, Stephan Heisinger

**Affiliations:** Department of Trauma Surgery, University Clinic of Orthopedics and Trauma Surgery, Medical University of Vienna, 1090 Vienna, Austria; Department of Trauma Surgery, University Clinic of Orthopedics and Trauma Surgery, Medical University of Vienna, 1090 Vienna, Austria; Section of Pediatric Trauma Surgery, Department of Trauma Surgery, University Clinic of Orthopedics and Trauma Surgery, Medical University of Vienna, 1090 Vienna, Austria; Department of Trauma Surgery, University Clinic of Orthopedics and Trauma Surgery, Medical University of Vienna, 1090 Vienna, Austria; Department of Trauma Surgery, University Clinic of Orthopedics and Trauma Surgery, Medical University of Vienna, 1090 Vienna, Austria; Department of Trauma Surgery, University Clinic of Orthopedics and Trauma Surgery, Medical University of Vienna, 1090 Vienna, Austria

**Keywords:** knee instability, pediatric knee joint, posterolateral corner of the knee, LCL injury

## Abstract

A 13-year-old struck by a car developed knee swelling and varus instability after treatment of a traumatic brain injury. Magnetic resonance imaging showed a displaced osseous avulsion of the lateral collateral ligament (LCL) from the fibular head with partial biceps femoris rupture, without meniscal or cruciate injury. After arthroscopic exclusion of intra-articular pathology, the fragment was reduced and fixed laterally using two suture anchors and a transosseous stitch, preserving the proximal fibular physis. At 1 year, the knee was stable and radiographs were normal. In displaced pediatric fibular LCL avulsions, prompt recognition and physeal-sparing suture-anchor fixation can restore stability, support early rehabilitation, and minimize growth-disturbance risk.

## Introduction

The lateral collateral ligament of the knee (LCL) is a strong ligament that runs along the lateral side of the knee. Proximally it is attached to the lateral femoral epicondyle and distally to the fibular head [1, 2]. Together with the structures of the posterolateral ligament complex (tendon of the popliteus muscle, popliteal arcuate ligament, popliteofibular ligament, posterior third of the lateral joint capsule, tendon of the biceps femoris muscle, and tendon of the plantaris muscle), the LCL provides static and dynamic stabilization of the knee joint and counteracts varus stress in particular [3]. Isolated injuries of the LCL can occur as ligamentous [4] or as an avulsion fracture of the fibular head [5] or the lateral femoral epicondyle [6]. Exact age- and population-related data on the incidence of isolated LCL injuries are lacking.

## Case report

Authors report on a 13-year-old male patient with an avulsion fracture of the LCL at the right fibular head. The type of injury, diagnosis, treatment, and outcome are described in the following.

### Medical history and diagnosis

**Figure 1:**
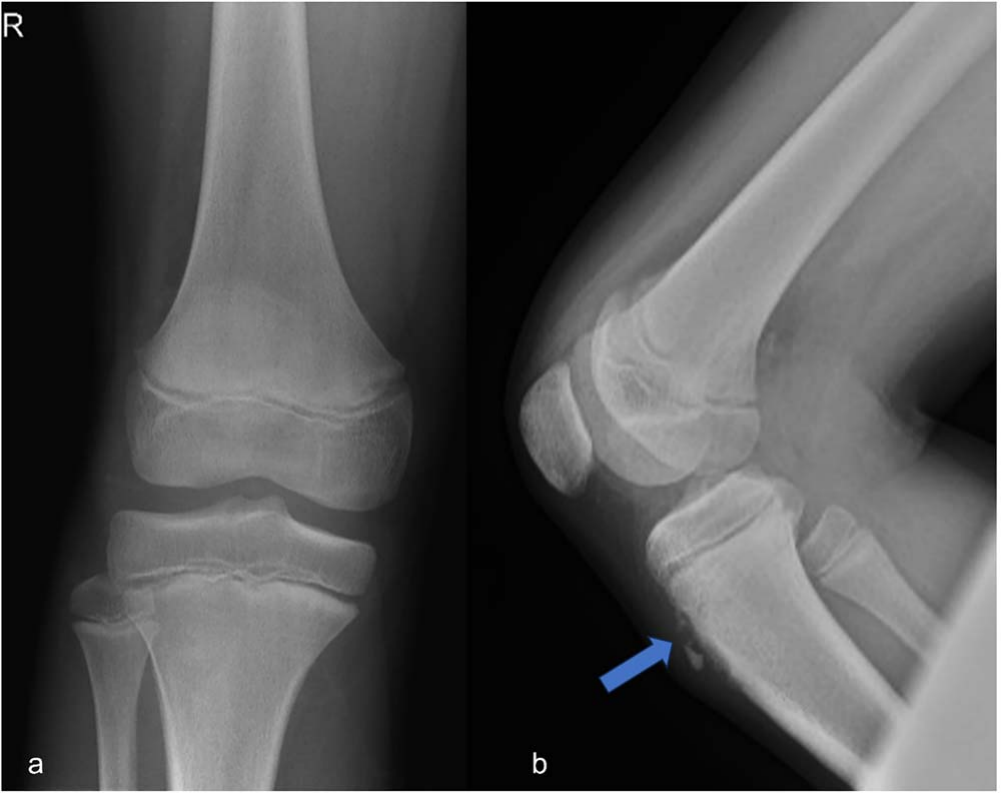
Conventional radiograph in the primary diagnosis. In the anteroposterior (a) and lateral (b) projection, there is no evidence of a fracture or epiphysiolysis. A secondary finding is an ossification disorder in the area of the tibial tuberosity as an expression of Osgood-Schlatter disease (arrow).

The patient was hit by a car traveling at around 40 km/h when crossing the road. More specifically the impact of the bumper on the right knee—with a fixed right foot—resulted in extensive varus stress on the knee joint. Initial management focused on the severe traumatic brain injury. The following day, a painful right lower extremity with significant swelling of the knee region and reduced range of motion (ROM) 0–5–40° as well as lateral instability at 30° of flexion were found. Sensation and perfusion were intact and there was no clinical evidence of compartment syndrome. The knee radiograph was otherwise unremarkable and age-appropriate, with only incidental findings consistent with symptomatic Osgood-Schlatter disease (Fig. 1a and b).

**Figure 2:**
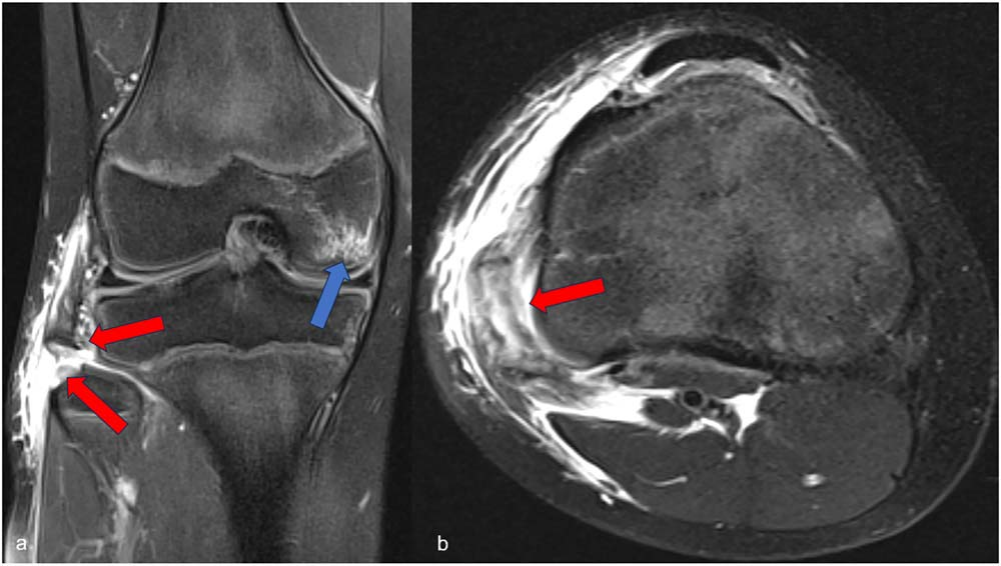
MRI of the right knee joint. (a) The coronal PD TSE FS section shows an osseous avulsion of the LCL at the tip of the fibular head (red arrows). On the medial side, a bone bruise in the medial femoral condyle is evident (blue arrow). Overall, the epiphyseal plates appear intact. (b) The axial PD TSE FS section shows the edematous, distended, ruptured LCL (red arrow).

The consecutive knee magnetic resonance imaging (MRI) (Siemens Aera, 1.5 T MRI, Germany) revealed an osseous avulsion of the LCL at the fibular head (Fig. 2a and b) with edematous distension of the lateral tendon of the biceps femoris muscle, the tendon of the plantaris muscle, the lateroventral border of the gastrocnemius muscle (caput laterale), and the tendon of the soleus muscle. A small bone bruise in the medial condyle of the femur was found without any further injuries. Computed tomography (CT) was not obtained, as MRI together with the clinical finding of varus instability provided sufficient information to establish the diagnosis, assess associated soft-tissue injury, and guide the choice of treatment. In this skeletally immature patient, additional CT imaging was therefore avoided to limit ionizing radiation exposure.

### Surgical treatment

In the case of an osseous avulsion of the LCL and an unstable knee, surgical treatment was indicated.

**Figure 3:**
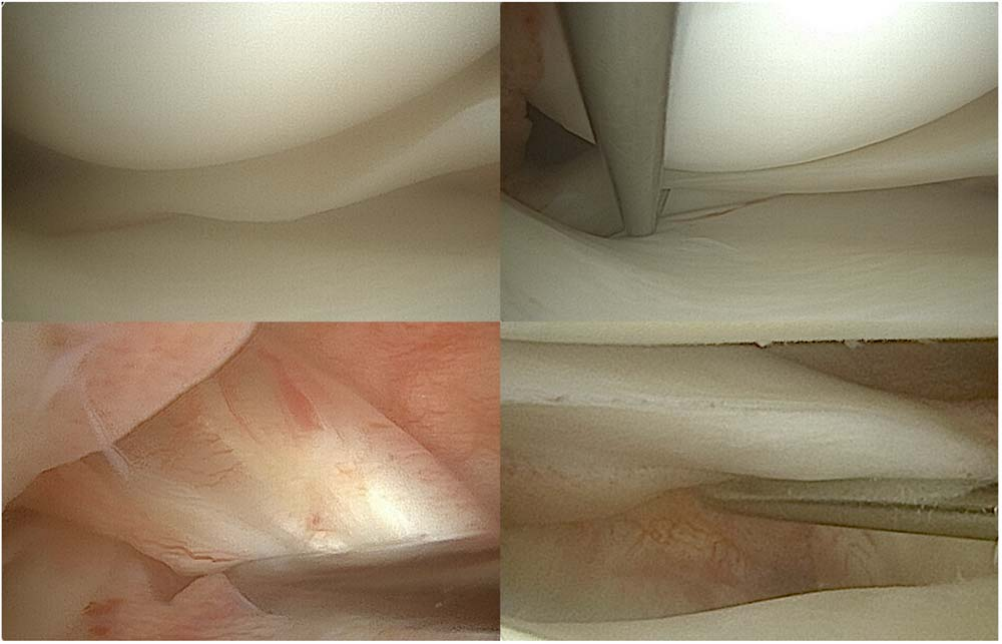
Diagnostic arthroscopy of the right knee. Representative arthroscopic views demonstrate no relevant intra-articular concomitant injuries: smooth articular cartilage surfaces, no meniscal tear (stable meniscus on probing), and no macroscopic abnormalities of the cruciate ligaments. Overall, the arthroscopic inspection was unremarkable, supporting an isolated extra-articular LCL avulsion injury.

Examination under general anesthesia confirmed lateral instability and showed negative clinical testing for other knee ligaments. At first a diagnostic arthroscopy was performed to exclude potential concomitant injuries, which revealed no abnormalities, in particular the posterolateral corner was unremarkable (Fig. 3). An approximately 8-cm longitudinal skin incision was made over the lateral aspect of the knee, centered over the fibular head. Meticulous dissection was carried through the subcutaneous tissue with careful hemostasis. The fibular head and the avulsion fracture were exposed and visualized. The common peroneal nerve was at risk during this dissection given its anatomic course dorsal to the biceps femoris tendon approximately 3 cm distal to the fibular styloid, and careful tissue handling was employed to protect neurovascular structures. After dissection and extensive irrigation, the LCL was secured with a FiberWire, which was further used to retain anatomical reduction manually. Two Corkscrew suture anchors (2.2 × 4 mm) were placed in the fracture bed within the fibular head epiphysis, directed from anterolateral to posteromedial to maximize bone stock and avoid the proximal fibular physis located approximately 5–10 mm distal to the fibular head. The thin cortical osseous fragment of the avulsed LCL was then sutured and fixed using FiberWire sutures passed through the anchors. To complete the reduction, a transosseous suture was applied, leaving the epiphysis intact. After reconstruction, the knee was stable on ligaments on all sides and flexion up to 90° was possible without any problems or any extension deficit. On closer inspection, the tendon of the biceps femoris muscle was partially ruptured, so it was subsequently reinserted as well with the aid of a Corkscrew anchor. The growth plate of the fibular head was not touched at any stage. The wound was closed after extensive irrigation without the insertion of a drain.

**Figure 4:**
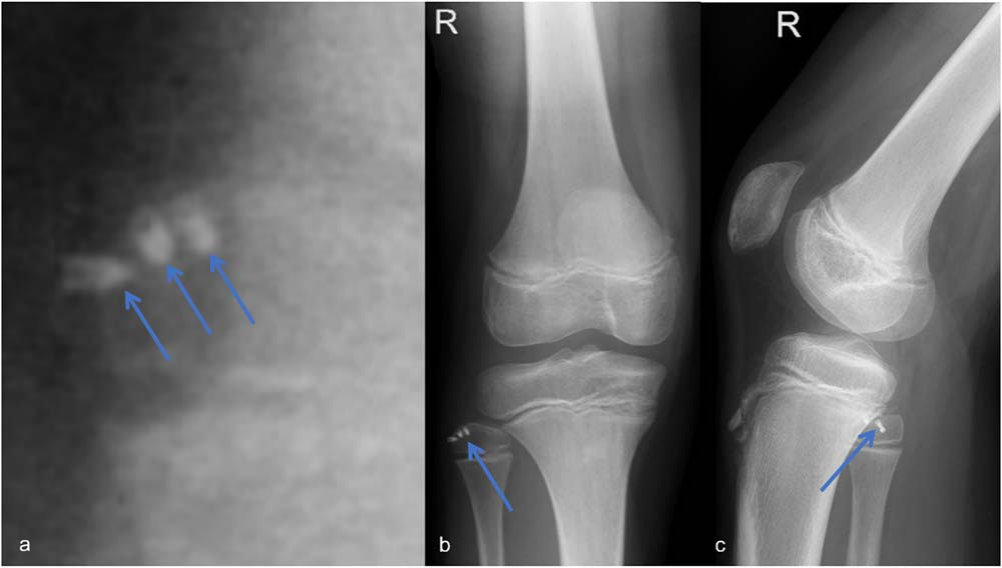
Intra- and postoperative radiographs. (a) In the magnified image, the corkscrews are located subcortically in the dorsolateral area of the fibular head (blue arrows). (b, c) The radiographs show continuous adaptation of the osseous avulsion fragment. The inserted corkscrew anchors in the fibular head (blue arrows) — two for the avulsed LCL fragment and one for the reinserted biceps femoris tendon — are at a clear distance from the growth plate.

The final X-ray showed a continuous refixation of the osseous LCL tendon avulsion with three dorsolaterally inserted Corkscrew anchors (Fig. 4a–c).

### Follow-up treatment and outcome

**Figure 5:**
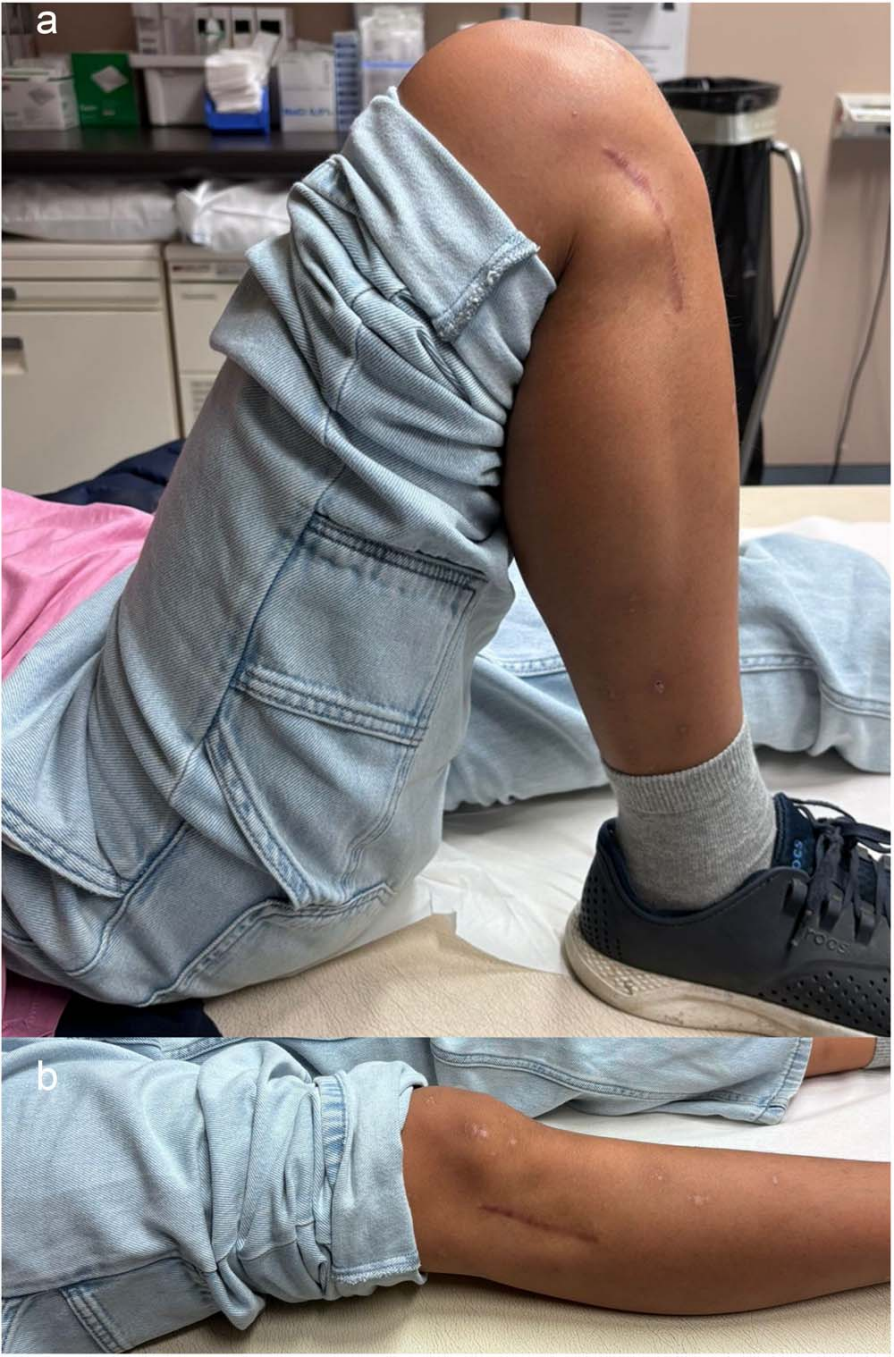
Clinical examination of the right knee. Physiological range of motion using the neutral-zero method: S (0–0–130°). No flexion (a) or extension (b) deficit. Stable to varus/valgus stress at 0° and 30° of flexion; anterior/posterior drawer unremarkable; Lachman and pivot-shift negative; meniscal tests (McMurray/Thessaly) negative. The lateral incision scar has healed without irritation.

**Figure 6:**
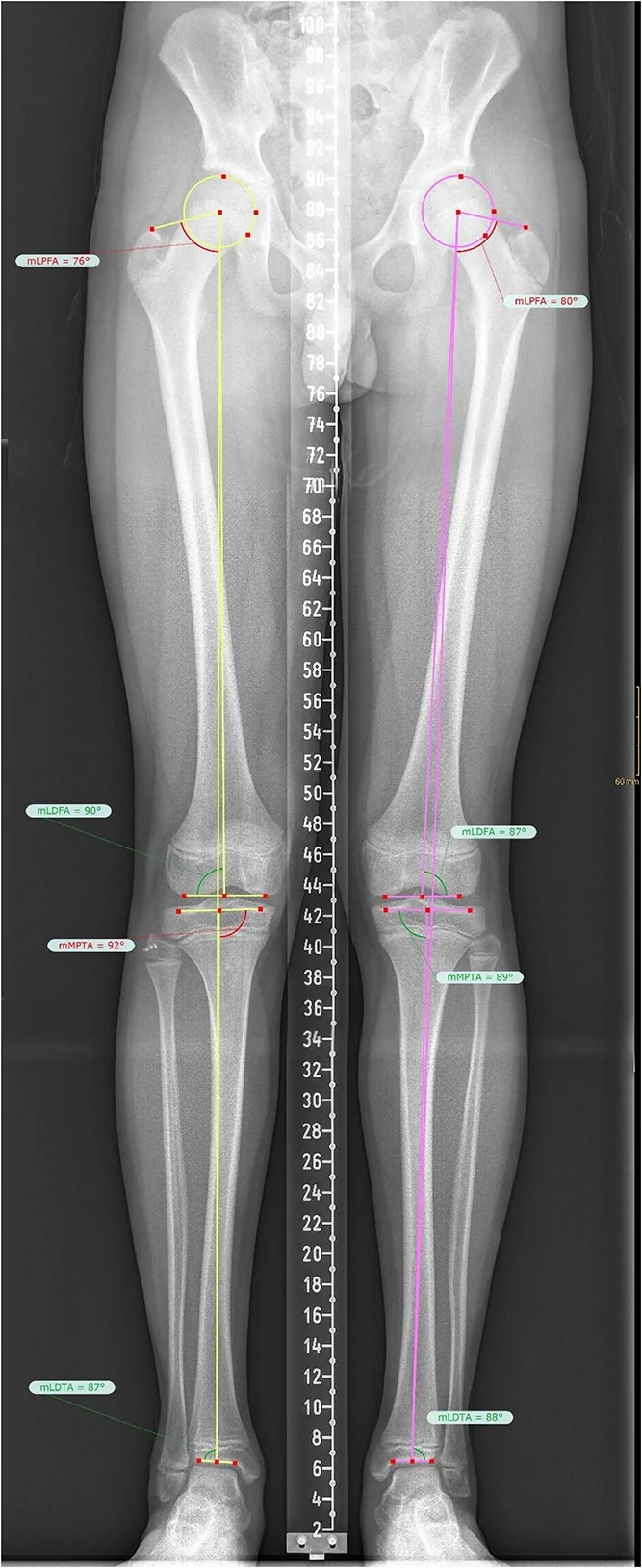
Full-length standing radiograph for leg-axis analysis (6 months post-op). Overall physiological alignment with a subtle side-to-side difference: HKA ≈ 180° bilaterally. Mikulicz line passes just lateral to the knee center—Right MAD 1 mm, left MAD 3 mm (lateral). Joint angles: mLPFA right (R): 76°, left (L): 80°; mLDFA R: 90°, L: 87°; mMPTA R: 92°, L: 89°; mLDTA R: 87°, L: 88°; JLCA R: 2°; L: 1°. Overall neutral alignment with a minimal valgus tendency on the left. Implant position normal. Measurements were performed using TraumaCad (Brainlab), version 2.5 (build 2.5.10.1001).

The patient was immobilized with a knee orthosis for a total of 6 weeks (initially 2 weeks ROM 0–0–30°, then 2 weeks ROM 0–0–60°, and finally 2 weeks ROM 0–0–90°) with partial weightbearing. In addition, physiotherapy was carried out continuously. At 9 weeks postoperatively, the knee was stable with a pain-free ROM of 0–0–130° (Fig. 5a and b). The lateral collateral ligament complex was stable under manual stress. The patient was able to return to recreational sports without restrictions after a further 3 months due to complete restoration of pain-free full ROM (0–0–130°), clinical ligament stability under varus stress at both full extension and 30° of flexion, absence of effusion, symmetric quadriceps and hamstring strength, and satisfactory performance in functional testing including single-leg hop symmetry. A whole-leg X-ray 6 months and 1 year postoperatively continued to show correct positioning of the implants without any accompanying growth disturbance or valgus/varus deformity (Fig. 6). At the final follow-up, the KOOS-Child overall score was 95/100, consistent with an uncomplicated pediatric healing course and restitutio ad integrum.

## Discussion

Isolated injuries to the LCL are very rare in adolescents with approximately 10% osseous involvement [7]. Most frequently caused by direct force application, sports injuries also involve combined vectors such as varus, extension, rotation, and translation stress, which can then lead to further ligamentous and osseous injuries in the posterolateral corner [8, 9]. Meniscal or cruciate ligament lesions are not usually found in this context [10], which could also be excluded in our patient by means of arthroscopy.

In patients without relevant distraction of the ligamentous and osseous structures and without significant instability, conservative treatment measures show satisfactory results [4, 11], this applies in particular to adolescents [10]. However, in the presence of significant varus instability (as in our patient), surgical treatment is indicated, as untreated chronic instability in children can lead to functional complaints with persistent pain and potentially leading to a growth disturbance or axial deviation. Harhaji *et al*. [5] achieved stable conditions in a 22-year-old American football player by refixing an osseous avulsion fracture of the proximal fibula using wire cerclage. As the growth plate was still open in our 13-year-old patient, careful refixation was performed using Corkscrews without traumatizing the growth plate. The patient was able to return to recreational sports without restrictions, and a whole-leg X-ray showed the correct position of the implants in the absence of axial deviations or growth disturbances.

## Conclusion

This case highlights the relevance of MRI imaging in addition to conventional X-ray to assess ligamentous, meniscal, and osseous lesions as well as a thorough clinical examination [8, 12]. Depending on the degree of ligamentous distraction and osseous dislocation, surgical repair should be performed in adolescents to stabilize the joint. Furthermore, it is important to avoid additional trauma to the growth plates during the procedure. Overall, we were able to achieve excellent results with our epiphyseal-sparing technique enabling the patient to return to recreational sports within 3 months.
